# Sclerosing Epithelioid Fibrosarcoma of the Thoracic Vertebrae: An Fairly Unusual Case Report With a Short Review of Literature

**DOI:** 10.3389/fmed.2022.833864

**Published:** 2022-06-28

**Authors:** Changhong Wei, Yili Ma, Dengqiang Wu, Xiaoyu Chen, Chunjun Li, Jun Chen, Sufang Zhou

**Affiliations:** ^1^Department of Pathology, Guangxi Medical University Cancer Hospital, Nanning, China; ^2^Guangxi Key Laboratory of Bio-Targeting Theranostics, National Center for International Research of Bio-Targeting Theranostics, Collaborative Innovation Center for Targeting Tumor Diagnosis and Therapy, Guangxi Medical University, Nanning, China; ^3^Department of Biochemistry and Molecular Biology, School of Pre-clinical Science, Guangxi Medical University, Nanning, China

**Keywords:** soft tissue sarcoma, sclerosing epithelioid fibrosarcoma, immunohistochemistry, fish, EWSR1

## Abstract

Sclerosing epithelioid fibrosarcoma (SEF) is a rare subtype of soft tissue tumors, and SEF originating from the side of the spine is even rarer. We report that a 28-year-old young woman suffered from chest pain and back pain for 3 years, and thereafter she went to see a doctor because her condition deteriorated. Enhanced CT showed that the right posterior upper chest wall mass invaded the adjacent bone, and the boundary between the lesion and the surrounding tissues was relatively clear. She then underwent posterior tumor removal surgery. The pathological examination confirmed the diagnosis of SEF. In histomorphology, the tumor displayed a typical epithelioid clear cell morphology, accompanied by extensive vitrification and fibrosis, which better helped to differentiate the tumor from low grade fibromyxoid sarcoma, solitary fibrous tumor and other entities. The immunohistochemical analysis showed a diffuse positive reaction to MUC4, a highly specific marker of SEF, which was detected by Immunohistochemistry (IHC), and fluorescence *in-situ* hybridization (FISH) confirmed that the *EWSR1* gene was rearranged, while the FUS gene was not rearranged. This is the first time that we have encountered such this rare case and thus report this case with updated literature related to this tumor.

## Introduction

Sclerosing epithelioid fibrosarcoma (SEF) is a rare type of soft tissue sarcoma, which predominantly occurs in the deep soft tissue tumors of the limbs and trunk (1, 2). There are only a few cases reported in other parts in China and abroad (3). This type of tumor displays epithelioid tumor cells arranged in sclerosing stroma as its typical histological characteristics. This tumor was first reported and named by Meis-Kindblom et al. in 1995 (4, 5), So far, more than 100 cases of this tumor have been reported in the literature, but sclerosing epithelioid fibrosarcoma of the paravertebral and chest wall has not been reported previously (6). Because it is relatively rare in clinical diagnosis and its performance is non-specific, so it can be often easily misdiagnosed as other benign and malignant tumors with epithelioid morphology and sclerosing stroma. This article aims to retrospectively analyze the rare SEF originating in the paravertebral and chest wall and review the literature, discuss its pathological characteristics and differential diagnosis, and thereby provide a basis for guiding clinical treatment.

## Case Presentation

**Figure 1:**
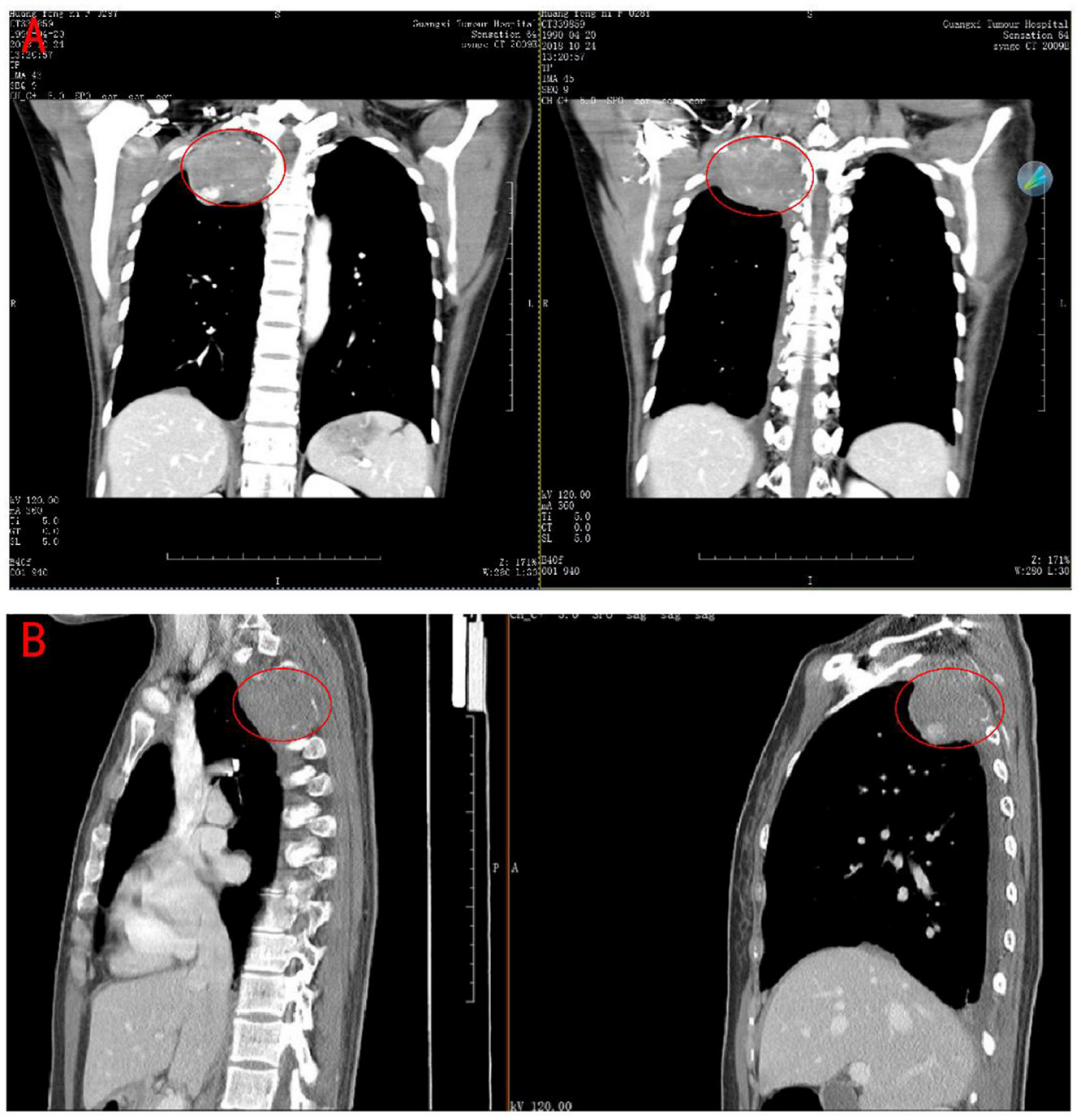
Computerized Tomography scan of the chest revealed a large tumor on the right posterior upper chest wall. **(A)** coronal plane. **(B)** sagittal plane.

A 28-year-old young female patient came to our hospital for the treatment. She had a 3-year medical history of chest pain on the right side. A CT scan outside the hospital found a mass in her lungs 7 days ago. The admission CT showed that there was a prominent mass on the right posterior upper chest wall invading the adjacent vertebral body but the boundary between the lesion and the surrounding tissues was relatively clear (Figures 1A,B). The laboratory examination did not reveal any specific findings. After completing other examinations, the clinician surgically removed the tumor. During the operation, it was observed that there was a tumor of about 8 ^*^ 7 ^*^ 5 cm on the right posterior chest wall at the position of the 1–4 posterior ribs. The cut surface of the tumor was fish-like, and it possesses a tough texture, poor mobility, and abundant blood supply, thus destroying the right posterior rib.

The specimens were thereafter fixed in 4% neutral buffered formaldehyde and then embedded in paraffin, sectioned routinely, stained with HE, and observed under a microscope. The specific experimental procedures were carried out in strict accordance with the instructions, and the control was established routinely. We used fluorescence *in situ* hybridization to detect the status of EWSR-1 and FUS genes in the tumor tissues. According to the previously reported protocol, EWSR-1 and FUS fragmentation probes were used for FISH. If more than 10% of the 100 cells analyzed show rearrangements, the tumor sample was considered as positive. The tumor was evaluated and scored by two independent investigators.

**Figure 2:**
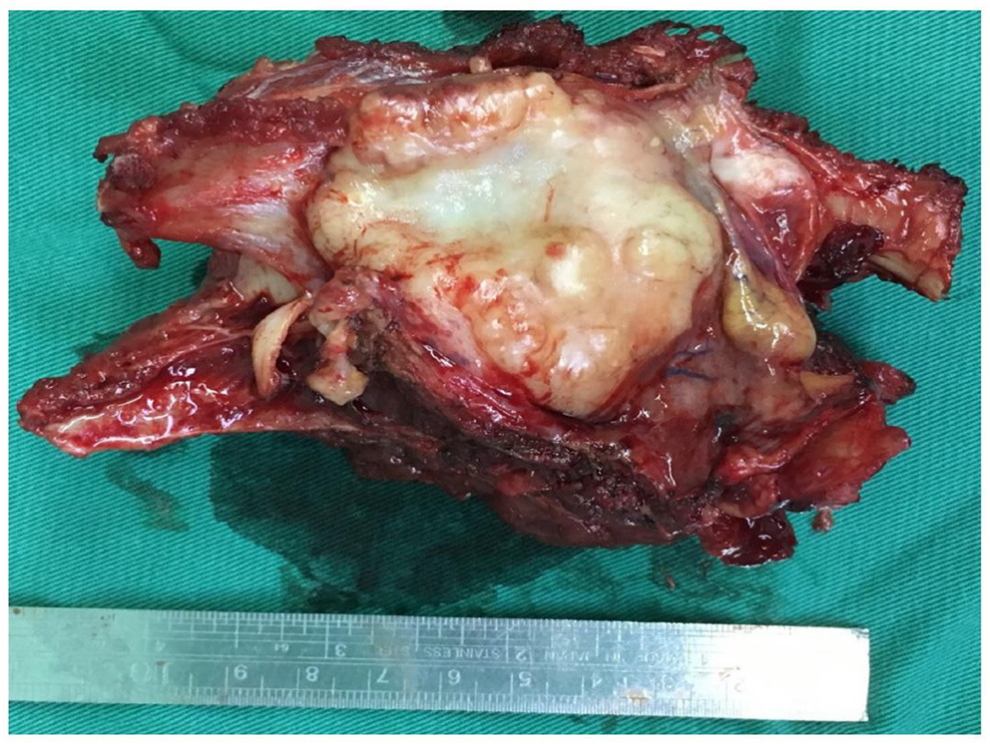
Gross photograph of the sectioned specimens from revealed a gray-yellow gray-brown foliated nodular tumor.

After clinical resection, a gray-yellow gray-brown foliated mass with a size of 8 × 7 × 5 cm was sent for the pathological evaluation. The surface of the mass appeared to be enveloped. The cut surface was grayish-yellow-white, solid, and tough, with mucinous degeneration in some specific areas (Figure 2).

**Figure 3:**
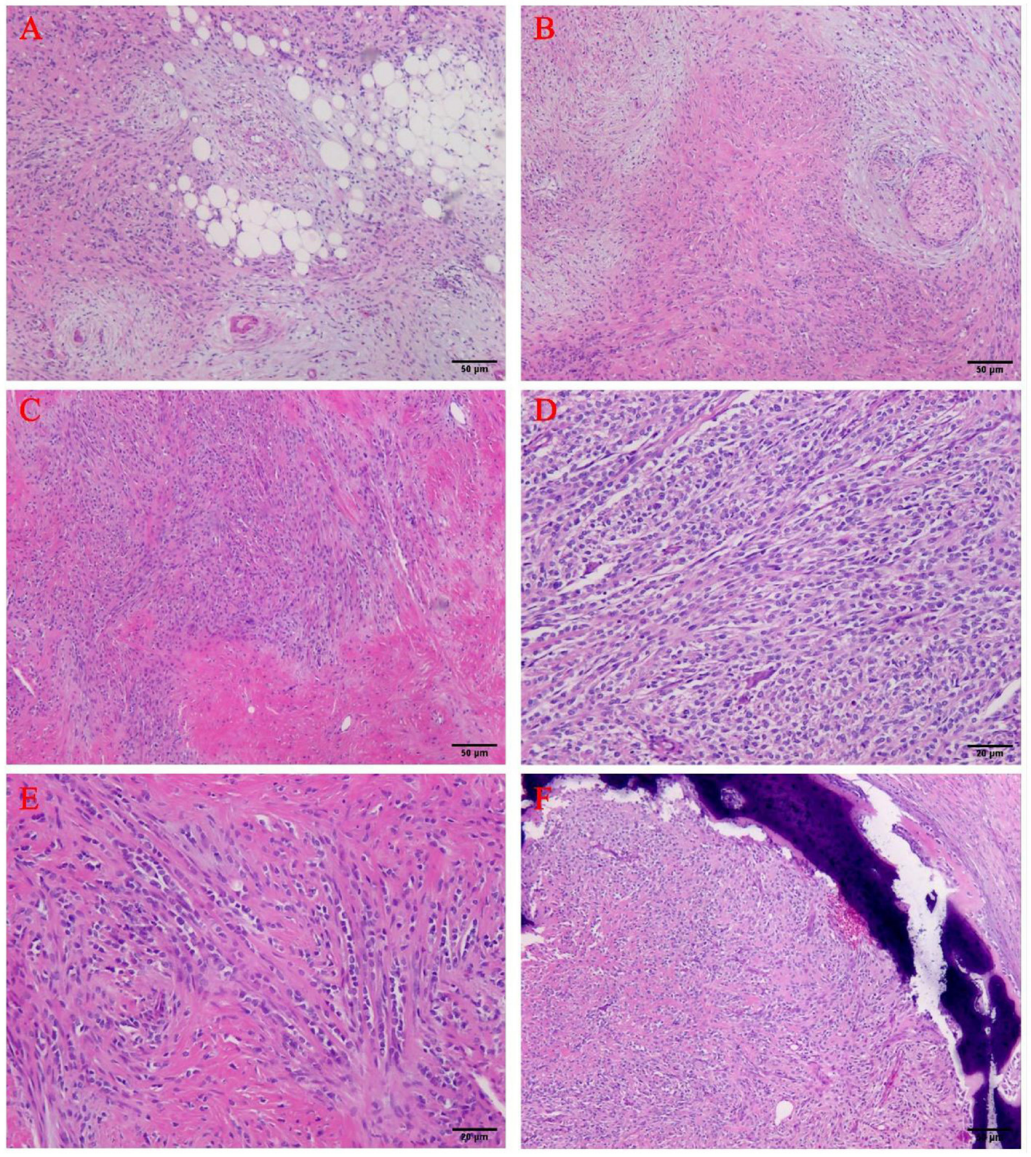
H&E staining results. **(A–C)** The tumor-rich areas, collagen fiber sclerosis areas and mucus areas were found to be staggered (40X). **(D,E)** The tumor cells were embedded in the fibrous matrix, and small and medium-sized epithelioid cells were arranged in cords and nests (100X). **(F)** The tumor invaded the bone of the spine (40X).

The boundary of the entire tumor was still clear. The tumor cells were abundant, and the morphology was relatively uniform. They were arranged in single, nested, cord-like, or sheet-like arrangements in the vitreous collagen interstitial, showed infiltrating growth, and the local bone tissue invasion can be seen. The stroma was composed of a large number of collagen fibers with obvious hyaline degeneration and occasional mucus-like areas. The tumor cells showed diverse morphologies and were spindle-shaped, short spindle-shaped, or irregular-shaped epithelioid cells. The tumor cell nuclei were oval, round, or polygonal, with sparse cytoplasm and rare mitotic images. A part of the area of the tumor showed that broad hyalinized collagen areas and mucinous degeneration-like areas were alternately arranged. In the peripheral part of the tumor, the cells were spindle-shaped and arranged in the bundles, exhibited fibromatosis-like images, and migrated with epithelial-like cells. Fatty changes could be seen locally in some specific areas (Figure 3).

**Figure 4:**
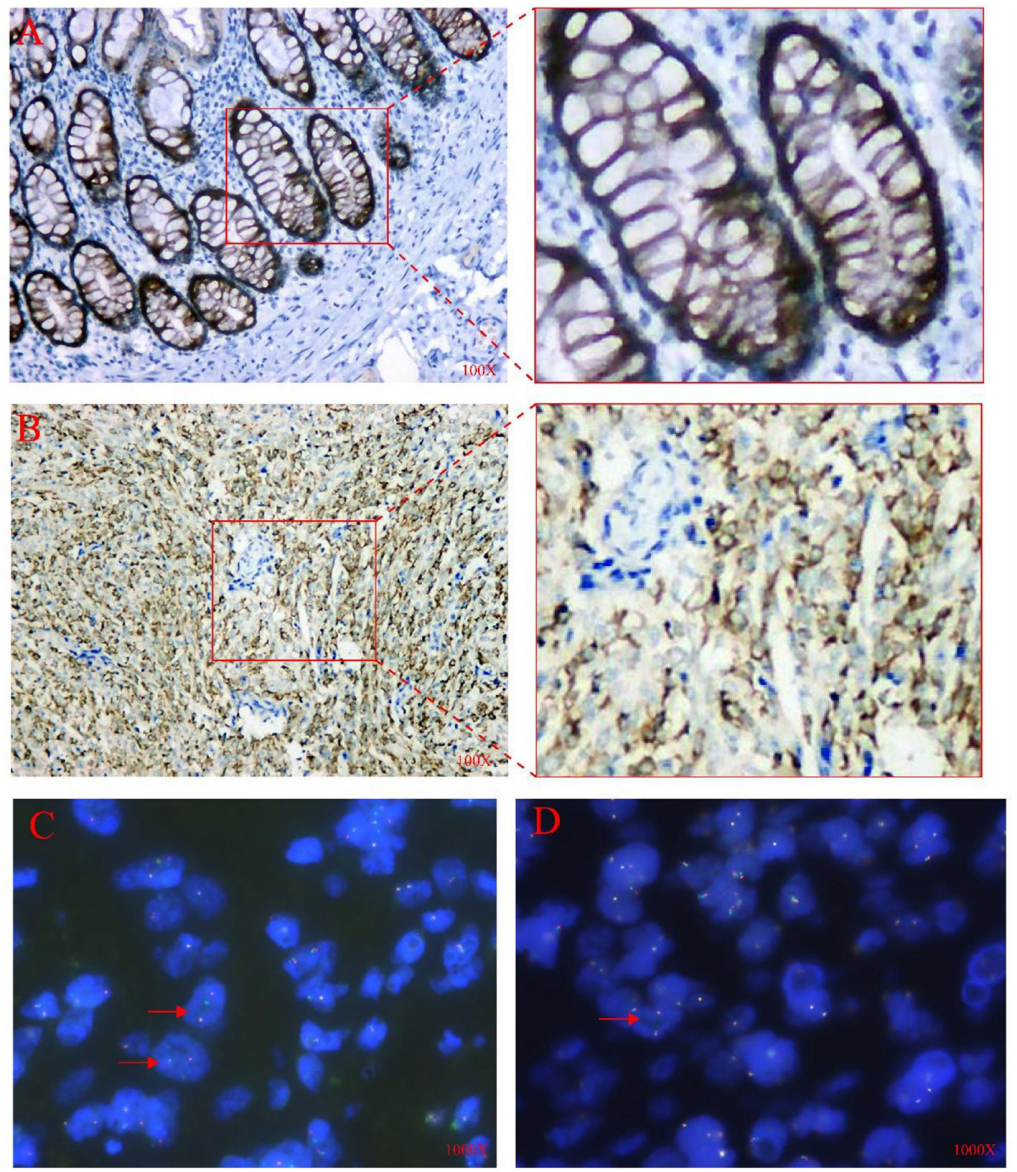
IHC and FISH analysis results. **(A)** Positive control for MUC4 showing normal (colon glands), 100X. **(B)** The tumor cells were diffusely positive for MUC4 in the cytoplasm,100X. **(C)** FISH analysis using the EWSR1 dual-color break-apart probe showed one red/green fused signal and one or two red split signals per nucleus in several tumor cells (shown by arrows),1000X. **(D)** FISH analysis using FUS dual-color break-apart probe revealed more than two red/green fused signals per nucleus in most tumor cells (shown by arrow), 1000X.

We did an immunohistochemical test for the tumor tissues, the immunohistochemical examination of the tumor showed positivity for Vimentin, MUC4, and CD99, while the other tumor markers such as CK, EMA, Calretinin (CR), WT1, D2-40, CD34, STAT6, CD99, β-catenin, S100, SMA, Desmin and SATB2 were negatively expressed, and Ki67 index as proliferation marker was about 10% (Figures 4A,B, **(A)** MUC4 expression in colon glands (positive control), **(B)** MUC4 expression in tumors).

The FISH analysis uses FUS and EWSR1 dual-color lysis probe to detect and analyze paraffin sections. For the EWSR1 lysis probe, among 200 different tumor cells, about 40% of the cells showed one fusion (red/green) signal and one or two red cleavage signals per nucleus. FISH results suggested that there was a non-interchangeable translocation involving the EWSR1 gene. On the contrary, for FUS fragmentation probes, the tumor cells showed 3–10 fusion (red/green) signals per nucleus. However, none of these were tested to determine their genetic partners or fusion breakpoints (Figures 4C,D).

Thereafter, we further used the next-generation sequencing technology based on targeted deep sequencing to detect the potential RNA sequence of this case specimen, the RNA sequencing was performed on an Illumina NextSeq platform. We found that there was presence of EWSR1-CREB3L1 fusion, which was composed of exon 9 of *EWSR1* and exon 6 of *CREB3L1*. However, there were no abnormal changes observed in the *FUS* gene.

## Discussion

SEF is a rare and special type of fibrosarcoma, which was first reported by Meis-Kindblom and other scholars in 1995, and there have been only few clinical reports since then. Most cases are located in the soft tissues of the trunk, upper and lower limbs, and head and neck. There are also reports that it can occur in intracranial, retroperitoneal, pelvic, bone, and ovarian areas (7). We report in this study that the tumor occurred in the posterior chest wall and side of the spine. We found that this site has not been identified previously in the domestic and foreign literature.

From the gross pathological specimens, the tumors were mostly lobulated or nodular in shape, with clear boundaries. The cut surface was gray and hard, with mucinous, cystic, and calcified areas, but necrosis was rare. The tumor cells in this case were small to medium in size, like epithelial, round, oval or polygonal, arranged in a cord, nest, sheet or alveolar shape and distributed in a large number of hyaloid collagen fibers. In the cell-rich area, a classic nodular growth pattern was observed. Tumor cells often infiltrated the surrounding soft tissue and bone. Besides, studies have found that SEF and some low-grade fibromyxoid sarcoma could be found in the similar areas. Some experts believed that there might be a possible genetic relationship between SEF and LGFMS which was first proposed by Antonescu et al. in 2001. This was confirmed by several groups subsequently in further studies and was further underlined by the strong immunohistochemical expression of MUC4 in both tumor types. In addition, hybrid lesions composed of both subtypes have been described repeatedly (8). In fact, SEF exhibited a more aggressive biological behavior than LGFMS and showed not be designated as low-grade sarcoma (9). Although these three tumors have a certain overlap in their morphology, each has a unique histological structure, which is obvious and intuitive to observe under a low power microscope. Most of the LGFMS and HSCTGR cells were found to be fusiform, with scattered mixed distribution of the sclerotic chrysanthemum clusters and focal epithelioid cell areas. The chrysanthemum cluster centers on hyalinized collagen can surround epithelioid fibroblasts in a radial arrangement, while SEF was found to be composed of round or epithelioid cells and the main feature noted was that of the interspersed collagen fibers of hyaline to form a network structure.

MUC4 is a relatively sensitive and specific tumor marker that can effectively distinguish SEF from other epithelioid soft tissue tumors. We tested the MUC4 protein in this case and found that the tumor cells were diffusely positive, which further confirmed our diagnosis. The expression of the tumor proliferation marker Ki-67 in this case was only about 10%, which suggested that the tumor was relatively less malignant.

At the molecular genetic level, it is found that SEF can have *FUS* gene rearrangement, but the probability of *FUS* gene rearrangement in SEF has been found to be significantly lower than the expression of *MUC4* (10, 11). A number of the previous studies have reported that some SEFs can possess the fusion of *EWSR1-CREB3L1* or *EWSR1-CREB3L2* genes. In our case, FISH detection also found that the EWSR1 gene EWSR1 gene has a rearrangement. At the same time, we also used the next-generation sequencing technology to detect the *FUS* gene but it was found that there was no abnormality (12, 13). These molecular genetic discoveries were helpful in the diagnosis of SEF, and also can provide a new way and basis for the pathological diagnosis of SEF (11). The *EWSR1-CREB3L1* gene fusion is the most commonly observed genetic abnormality in pure SEF, while the *FUS-CREB3L2* transcript is usually present in hybrid SEF/LGFMS (13, 14). Although low-grade fibromyxoid sarcoma confirmed by cytogenetics may have sclerosing epithelioid fibrosarcoma-like areas, FUS rearrangement is an important feature of hybrid SEF and is relatively rarely found in the pure sclerosing epithelioid fibrosarcoma. Low-grade fibromyxoid sarcoma is primarily characterized by fibrous areas composed of spindle-shaped fibroblast-like cells and mucous-like areas, which have repeated translocations involving *FUS* (16p11), *CREB3L2* (7q32–34), or *CREB3L1* (7p11) (15, 16). Pure sclerosing epithelioid fibrosarcoma (tumor lacking a recognizable low-grade fibroid sarcoma-like area) usually do not have *FUS* gene rearrangement. Moreover, some cases of sclerosing epithelioid fibrosarcoma may represent low-grade fibromyxoid sarcoma and might have obvious sclerosing epithelioid fibrosarcoma-like area (may be considered to be a low-grade fibromyxoid sarcoma in sclerosing epithelioid fibrosarcoma with *FUS* rearrangement) (17).

Because SEF is rich in epithelioid cells and spindle cells, it is often misdiagnosed as cancer or other types of spindle cell sarcoma. Therefore, the differential diagnosis from other soft tissue tumors is particularly important. The most common diagnosis in our daily work is mainly related to differentiation of low-grade fibromyxoid sarcoma (LGFMS) (18, 19). Moreover, SEF and LGFMS have a certain overlap in their molecular characteristics, and both of them can be fused with *EWSR1-CREB3L1* or *EWSR1-CREB3L2* (20). This shows that the two tumors are related, but 100% of LGFMS cases are accompanied by *FUS* gene rearrangement and translocation. LGFMS often exhibits alternately distributed collagen-like and mucous-like areas, mainly collagen-like areas, usually without the presence of the characteristics of epithelioid cells. The mesenchyme of SEF may also have mucinous degeneration, but it is also characterized by obvious sclerosis of the mesenchyme and the inclusion of round or polygonal epithelioid tumor cells arranged in a cord-like arrangement (14). It was found that the probability of SEF with FUS gene rearrangement is low, usually accompanied by *EWSR1* gene rearrangement.

Most experts consider SEF to be a low-grade fibrosarcoma as compared to fibrosarcoma, but its recurrence rate, metastasis rate and fatality rate will gradually increase over time (21). There is no standard systemic treatment plan available for SEF currently and the main treatment consists of extended surgical resection. At the same time, proper attention should also be paid to the regular follow-up of patients. The patient in this case was in good condition after surgical resection and has been followed up for more than a year and no signs of recurrence or metastasis were found. We need more time to observe her condition to determine whether there are any significant changes.

## Data Availability Statement

The raw data supporting the conclusions of this article will be made available by the authors, without undue reservation.

## Ethics Statement

The studies involving human participants were reviewed and approved by the Biomedical Research Ethics Committee of the Affiliated Cancer Hospital of Guangxi Medical University. The patients/participants provided their written informed consent to participate in this study. Written informed consent was obtained from the individual(s) for the publication of any potentially identifiable images or data included in this article.

## Author Contributions

JC and SZ: read and approved the final manuscript. CW: performed the writing of the manuscript. DW and YM: organized the material and helped with the analysis. XC: helped with analysis and constructive discussion. CL: performed the pathological analysis. All authors contributed to the article and approved the submitted version.

## Funding

This work was supported by the Guangxi Science and Technology Research Base and Talent-specific Project (AD18126021), self-funded scientific research project of Guangxi Zhuang Autonomous region Health Commission (Z20190572 and Z20210959), and Key Laboratory of the Ministry of Education Project for Early Prevention and Treatment of Regional High-risk Tumors (GKE-ZZ202007).

## Conflict of Interest

The authors declare that the research was conducted in the absence of any commercial or financial relationships that could be construed as a potential conflict of interest.

## Publisher's Note

All claims expressed in this article are solely those of the authors and do not necessarily represent those of their affiliated organizations, or those of the publisher, the editors and the reviewers. Any product that may be evaluated in this article, or claim that may be made by its manufacturer, is not guaranteed or endorsed by the publisher.

## Abbreviations

CK: cytokeratin
CR: Calretinin.
